# The Insulin-Like Growth Factor System in the Long-Lived Naked Mole-Rat

**DOI:** 10.1371/journal.pone.0145587

**Published:** 2015-12-22

**Authors:** Malene Brohus, Vera Gorbunova, Chris G. Faulkes, Michael T. Overgaard, Cheryl A. Conover

**Affiliations:** 1 The Division of Endocrinology, Mayo Clinic, 200 First Street SW, Rochester, Minnesota 55905, United States of America; 2 University of Rochester, Department of Biology, 434 Hutchinson Hall, River Campus, Rochester, New York, 14627, United States of America; 3 School of Biological and Chemical Sciences, Queen Mary University of London, Mile End Road, London E1 4NS, United Kingdom; 4 Department of Chemistry and Bioscience, Aalborg University, Frederik Bajers Vej 7H, DK-9220 Aalborg Oe, Denmark; The Scripps Research Institute, Scripps Florida, UNITED STATES

## Abstract

Naked mole-rats (*Heterocephalus glaber*) (NMRs) are the longest living rodents known. They show negligible senescence, and are resistant to cancers and certain damaging effects associated with aging. The insulin-like growth factors (IGFs) have pluripotent actions, influencing growth processes in virtually every system of the body. They are established contributors to the aging process, confirmed by the demonstration that decreased IGF signaling results in life-extending effects in a variety of species. The IGFs are likewise involved in progression of cancers by mediating survival signals in malignant cells. This report presents a full characterization of the IGF system in the NMR: ligands, receptors, IGF binding proteins (IGFBPs), and IGFBP proteases. A particular emphasis was placed on the IGFBP protease, pregnancy-associated plasma protein-A (PAPP-A), shown to be an important lifespan modulator in mice. Comparisons of IGF-related genes in the NMR with human and murine sequences indicated no major differences in essential parts of the IGF system, including PAPP-A. The protease was shown to possess an intact active site despite the report of a contradictory genome sequence. Furthermore, PAPP-A was expressed and translated in NMRs cells and retained IGF-dependent proteolytic activity towards IGFBP-4 and IGF-independent activity towards IGFBP-5. However, experimental data suggest differential regulatory mechanisms for PAPP-A expression in NMRs than those described in humans and mice. This overall description of the IGF system in the NMR represents an initial step towards elucidating the complex molecular mechanisms underlying longevity, and how these animals have evolved to ensure a delayed and healthy aging process.

## Introduction

Aging can be described as the inevitable process of gradual decreases in physiological and biochemical functions, which are prominent in single cells as well as in whole organisms, and with death as the ultimate outcome. While external factors may contribute to extended or shortened lifespan by influencing endogenous effects, they are not themselves direct determinants of maximum lifespan. In line with the deterioration of physiological functions, the prevalence of detrimental outcomes in the form of age-related diseases is simultaneously growing. In the attempt to avoid these undesirable outcomes, extensive work has been carried out trying to describe the complex mechanisms underlying aging [1–4].

Due to the labor-intensive and time-consuming task of delineating aging mechanisms, short-lived animal models are typically used to describe human conditions. However, humans age slower than predicted, based on the common correlation between body mass and maximum lifespan. For this reason naturally long-lived animal models may be more suitable for delineating mechanisms of aging pertinent to humans [5, 6]. A promising species that meets this criteria is the longest living rodent known, the naked mole-rat (*Heterocephalus glaber*) (NMR) which lives 5- to 10-times longer than predicted for their body mass of a mouse [2, 7]. It shows negligible senescence and is resistant to certain damaging effects associated with aging, including oxidative stress [2]. Interestingly, no incidence of cancer has been observed in a NMR colony of more than 800 animals or in a large zoo population of NMRs [2, 8, 9]. This may be one of the most remarkable features described for this species and highly relevant to healthy human aging. The challenges ahead are to delineate underlying mechanisms. The recently published NMR genome [10] prepares the way for more thorough analyses. For this study, we chose to investigate the insulin-like growth factor (IGF) system.

The IGFs, through activation of transmembrane IGF receptors (IGF-R), have pluripotent actions, influencing growth processes in virtually every system of the body [11]. They are established contributors to the aging process, confirmed by the demonstration that decreased IGF signaling results in life-extending effects in a variety of species [12]. The IGFs are likewise involved in progression of multiple types of cancer [13]. Thus, the phenotypic traits of NMRs resemble those of organisms with impaired IGF signaling. Interestingly, IGF-IR protein levels in the brain show negative correlation with maximum lifespan across 16 rodent species, with the NMR having lower IGF-IR levels than the other rodents [5].

The bioactivity of IGFs is regulated by six high-affinity IGF binding proteins (IGFBPs) which can undergo a variety of post-translational modifications [14, 15]. While intact IGFBPs regulate IGFs mostly by sequestration, specific IGFBP proteases decrease IGF binding affinity and hence increase IGF bioavailability. One such protease is the metalloprotease, pregnancy-associated plasma protein-A (PAPP-A), which has also been proven to play a significant role in longevity, as PAPP-A knock-out mice live 30–40% longer than wild-type littermates [16]. In addition, these mice display reduced incidence and delayed occurrence of malignant neoplasias. Interestingly, the published NMR genome [10] suggested mutations in the active site of PAPP-A that would be detrimental to its proteolytic activity.

Thus, the aim of this study was to characterize the IGF system in the NMR, both at the genetic and protein level, with an emphasis on PAPP-A.

## Results and Discussion

### Sequence studies on IGF components in the NMR

We carried out a bioinformatic analysis for identifying components of the IGF system in the NMR that could potentially underlie its longevity and improved aging process. Such analyses enable a relatively fast overview of differences that may be relevant to analyze in detail experimentally. These studies are made possible for this species due to the two published genome sequences available [6, 10]. Based on our annotations of the genomic sequences, Table 1 lists the number of exons identified by BLAST analyses using data from the two available genome assemblies, and the conservation of protein sequences between humans, mice, and NMRs. Generally, mature NMR protein sequences resemble human more than murine sequences even though the similarities between all three species are relatively high for all components.

**Table 1:** Details of the annotated IGF components in the NMR.

Name	Number of exons identified	Sequence coverage (NMR/human/mouse)	Sequence identity (%) (human/mouse)
IGF-1	3	70/70/70	98.6%/94.3%
IGF-2	3	67/67/67	100.0%/91.0%
IGF-1R	21	1370/1367/1373	97.6%/95.8%
IGF-2R	48	2491/2491/2483	83.7%/82.3%
IGFBP1	4	247/233/247	68.8%/81.0%
IGFBP2	4	290/290/272	91.4%/83.1%
IGFBP3	4	243/264/265	84.0%/87.2%
IGFBP4	4	237/237/233	91.1%/87.8%
IGFBP5	4	252/252/252	97.2%/93.7%
IGFBP6	4	213/213/210	76.7%/66.7%
PAPP-A	22	1549/1547/1546	93.1%/92.6%

Sequence coverage of mature proteins is indicated by the lengths of NMR, human, and murine sequences. Percent identity is calculated using mature human and mouse protein sequences as references separated by /. Regions with lack of coverage were disregarded when calculating percent identity.

We used previously published data on amino acid residues and specific areas or motifs that are of importance in complex formation, as well as for other essential functions related to the proteins analyzed, to predict any functional or structural alterations in the NMR IGF system components. Residues demonstrated to make direct contacts to binding partners, but also indirect e.g. by stabilizing the structure around an important binding pocket, were analyzed. The analyses were done using human and murine sequences for comparison. Murine sequences are used to provide stronger evidence of NMR variations. At the same time, the use of murine sequences helps to indicate if a variation between human and NMR holds value. No observable variation between mouse and NMR indicates that the protein of interest most likely is functional despite deviation from the human counterpart. Hence, in the following analyses, residues of importance are indicated by arrows in mature protein sequences. Important areas are enclosed within a colored square. Additional features and sites of interest are described in each figure legend.

**Fig 1:**
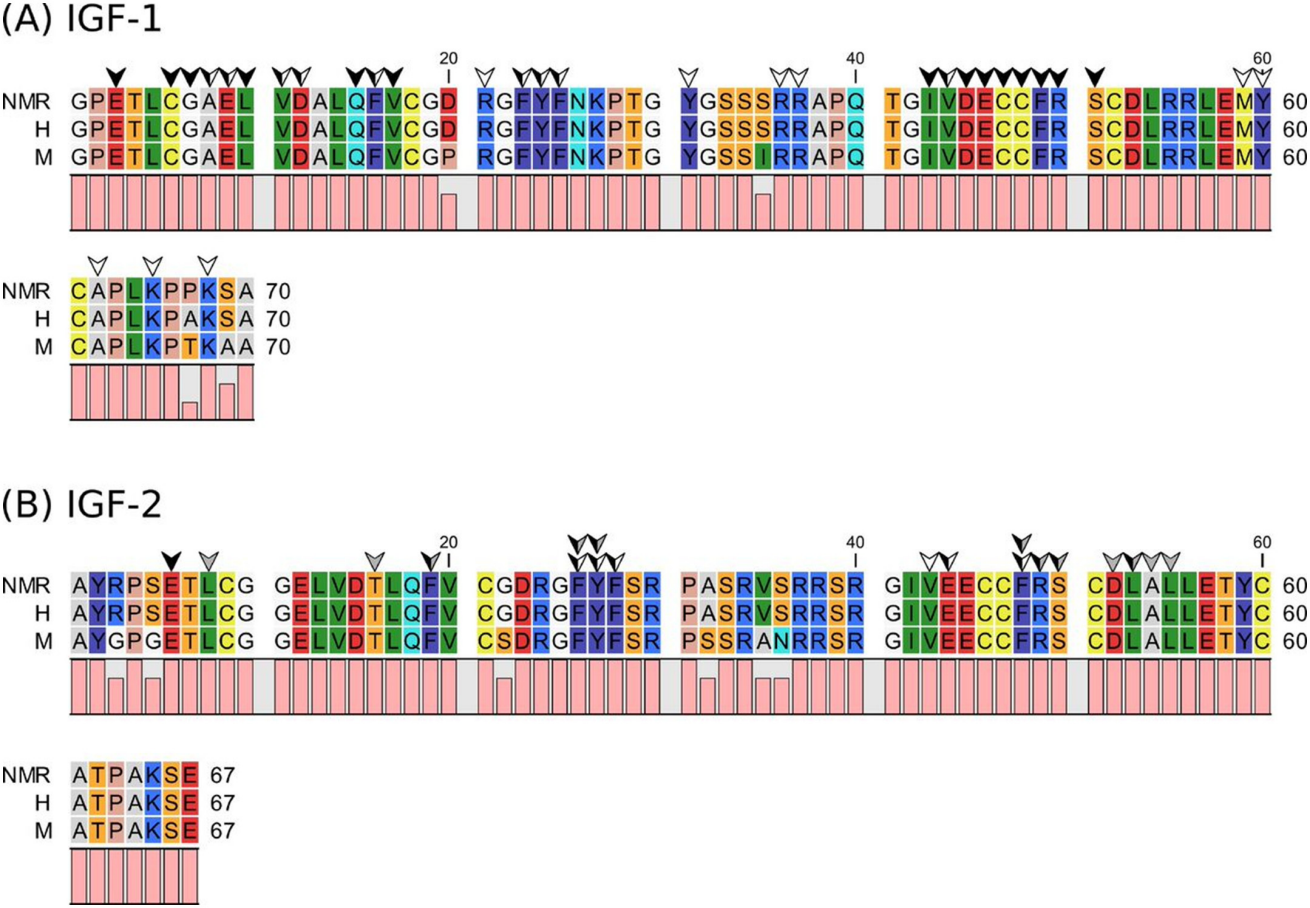
Sequence alignment of NMR, human, and mouse IGF-1 (A) and IGF-2 (B). White arrows indicate residues interacting with the IGF1-R, grey arrows indicate residues interacting with the IGF-2R, and black arrows indicate residues binding to the IGFBPs. Dual-colored arrows indicate residues that overlap in binding to the IGFBPs and IGF-1R or IGF-2R.

Mature NMR IGF-2 is 100% identical to hIGF-2 and NMR IGF-1 is 98.6% identical to hIGF-1 (Fig 1), with only one residue differing [17]. The one variant residue in IGF-1 (residue 67 is a proline in the NMR and an alanine in human) is not conserved between either species and is not an established binding determinant (Denley *et al*. 2005). Therefore, no strong evidence indicates that the NMR IGFs themselves would cause decreased or abrogated binding to their interacting partners.

**Fig 2:**
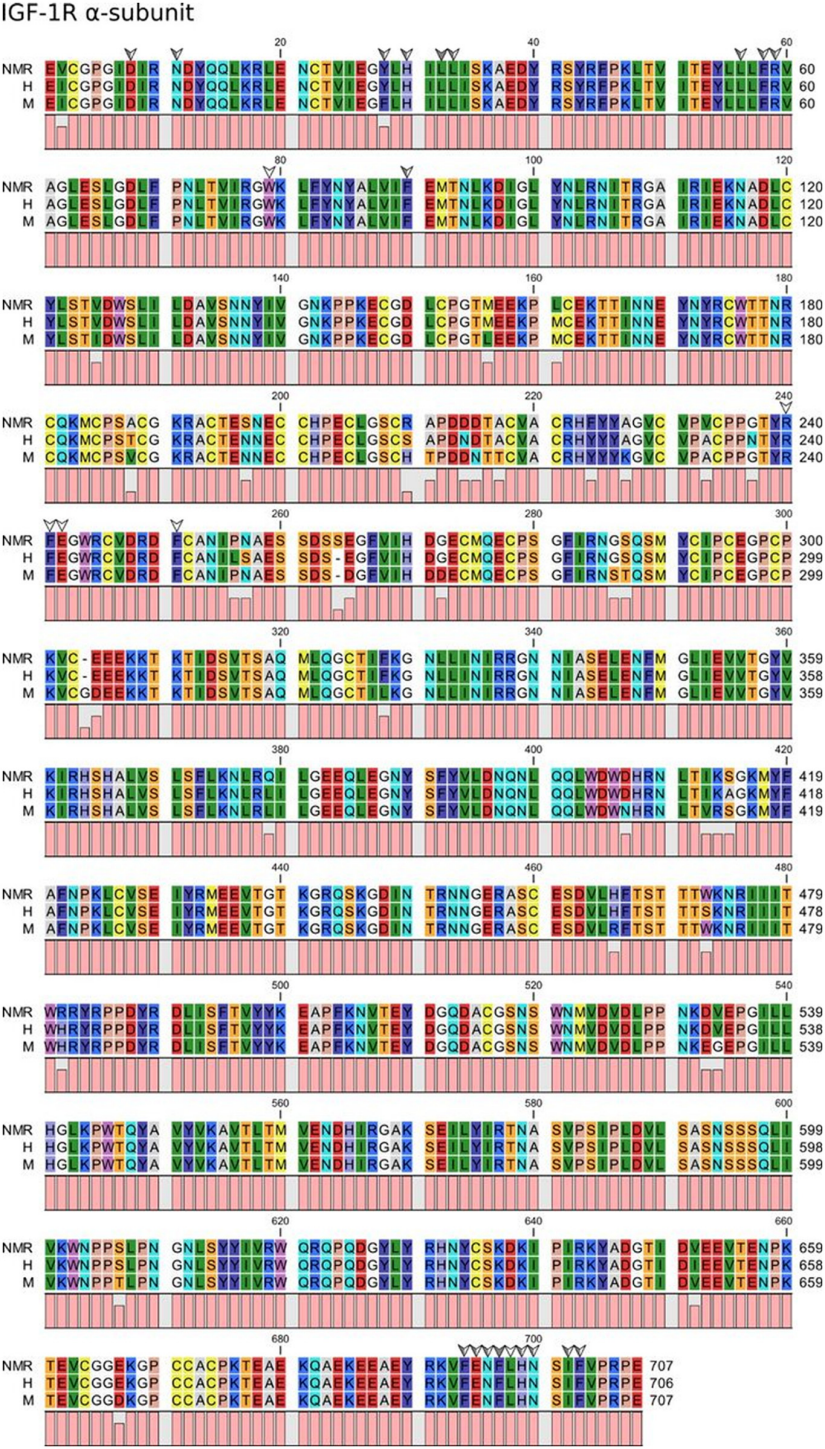
Sequence alignment of NMR, human, and mouse IGF-1R α-subunit and β-subunit. White arrows indicate residues interacting with IGF-1, grey arrows indicate residues interacting with IGF-2, and dual-colored arrows indicate residues that overlap in binding to IGF-1 and IGF-2.

**Fig 3:**
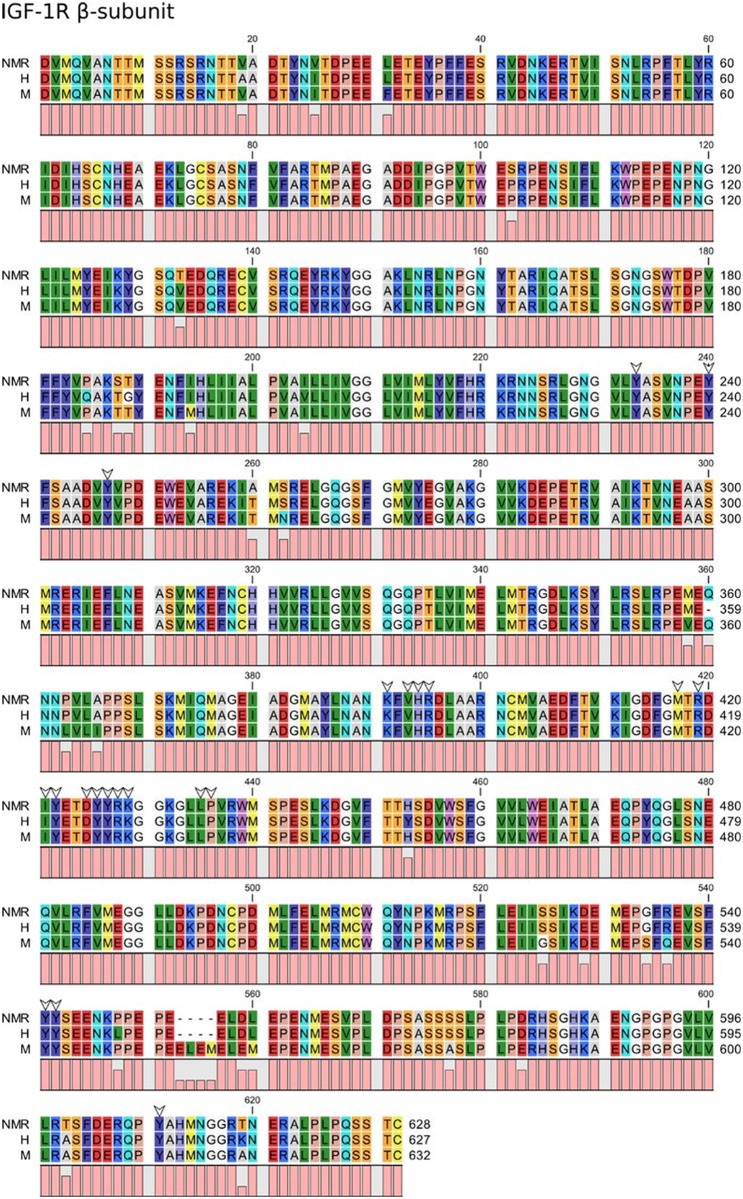
Sequence alignment of NMR, human, and mouse IGF-1R β-subunit. White arrows indicate residues important for IGF signaling by IGF-1R.

Structural variations may yet be present in important residues in the cognate IGF receptors, which could lead to impairment of IGF actions. However, on the IGF-1R side, the highlighted binding determinants are 100% conserved between all species considered. This applies both to the interacting residues in the ligand-binding α-subunit (Fig 2) and in the signal-mediating β-subunit (Fig 3), including all the residues that constitute the auto-inhibitory activation loop [17–20]. The overall high degree of conservation clearly illustrates the vital functions that belong to this protein. Several tyrosine residues in the β-subunit may be involved in mediating IGF signaling. All but one of the total 26 tyrosine residues are 100% conserved between all species. Tyr1162 in the human sequence is a histidine residue in both the murine and the NMR sequence, suggesting that even though this residue is located in the tyrosine kinase domain of the β-subunit, it probably has no particular importance in mediating IGF actions. In addition, Davies *et al*. [21] reported little sequence variation in the transmembrane domain of IGF-1R between NMR and mice.

As the IGF-2R is mainly relevant for IGF-2 in an IGF-physiological regard, only residues implicated in IGF-2:IGF-2R association are emphasized [22]. All of these are 100% conserved between human, mouse, and NMR (S1 Fig), suggesting that the NMR IGF-2R has evolved and retained the ability to sequester IGF-2.

**Fig 4:**
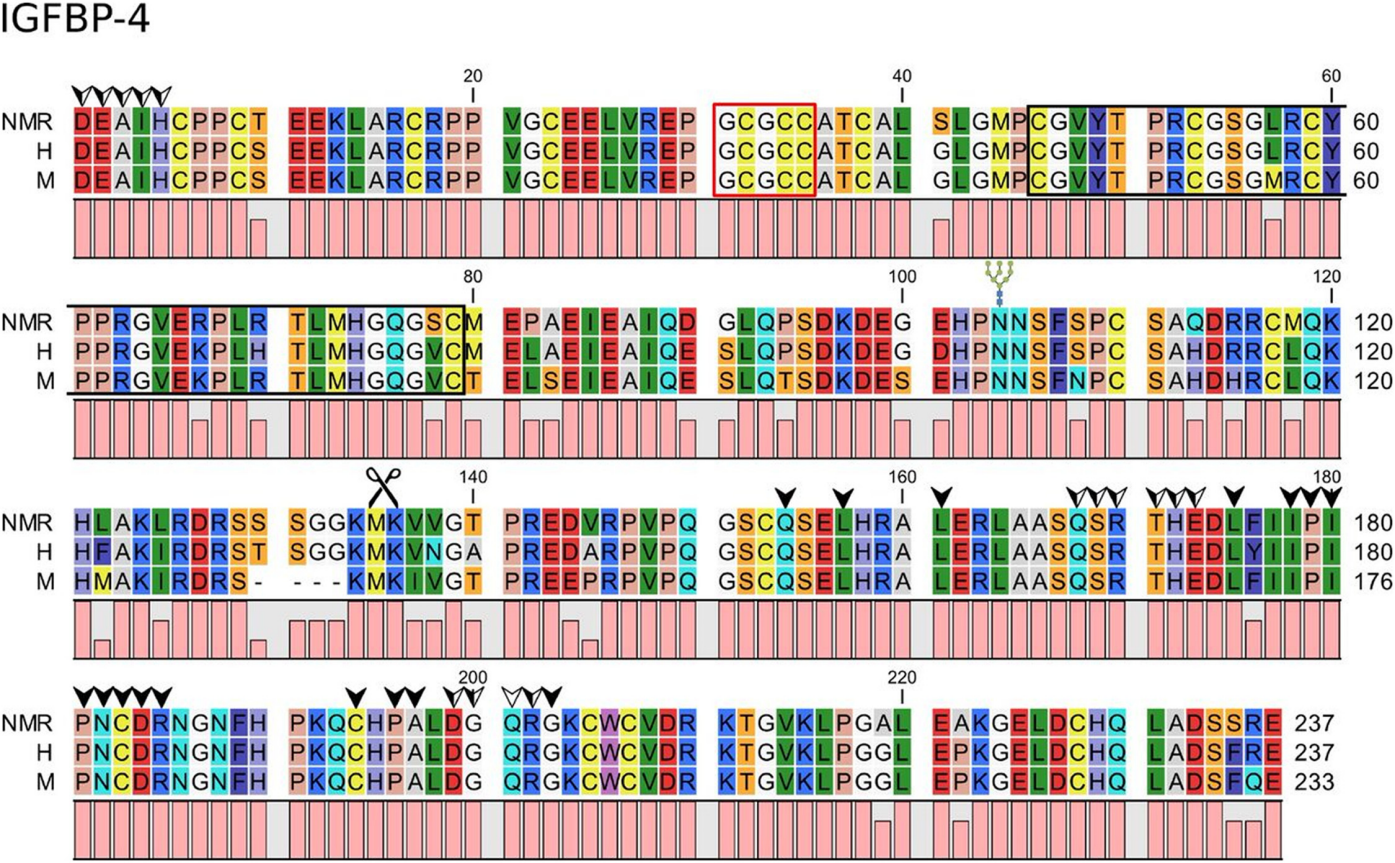
Sequence alignment of NMR, human, and mouse IGFBP-4. The N-terminal GCGCC motif is enclosed within a red square. The high-affinity N-terminal binding site is enclosed within a black square. Glycosylation sites are indicated by a sugar branch. The PAPP-A proteolytic site is indicated by scissors. White arrows indicate residues interacting with other parts of IGFBP-4, black arrows indicate residues involved in binding to IGF-1, and dual-colored arrows indicate residues that overlap in binding internally and to IGF-1.

For the IGFBPs [14, 17, 23–25], all cysteine residues in all IGFBPs are conserved, suggesting that the overall structural integrity is retained in the NMR IGFBPs (Fig 4, S2–S6 Figs). However, the 11th cysteine of human and murine IGFBP-1 (Cys61) is a glycine according to one of the NMR genome sequences (Accession: AFSB00000000) (S2 Fig). Because the cysteines are crucial for IGFBP structure and because the other genome sequence (Accession: AHKG00000000) does not contain this variation, we suggest that this database variation is most likely due to sequencing errors.

The compact N-terminal high-affinity IGF-binding region between the 9th and 12th cysteines (7th and 10th cysteine in IGFBP-6) is indicated by a black square in all IGFBPs (Fig 4, S2–S6 Figs). Differences are evident in this region for several of the IGFBPs. It is, however, difficult to tell if or to what extent the variations contribute to IGFBP binding of the IGFs. IGFBP-4 is the only IGFBP for which a high resolution structure of the ternary complex with both the N- and C-terminal domains bound to IGF-1 exists. Hence, residues implicated in this specific association will be emphasized (Fig 4). Residues where either the human or murine sequence is identical to the NMR counterpart can be ruled out as residues contributing to NMR-specific phenotypic traits. However, it may be relevant to look at those residues that vary between all three species as well as NMR residues that vary from identical human and murine sequences. In the compact region of IGFBP-4 (Fig 4), the NMR residues at Lys67 and Val78 differ from identical human and murine residues. NMR Arg67 retains a basic side-group and therefore does not result in changed functional properties of the amino acid compared to the corresponding human and murine basic lysine residue. On the other hand, the polar NMR Ser78 residue may confer different chemical properties and structural restraints than the murine and human hydrophobic valine.

Functional motifs, such as integrin and heparin binding domains, are largely conserved between humans, mice, and NMRs. However, some of the post-translationally modified residues show variations, which may indicate that some regulatory mechanisms of the IGFBPs vary between NMRs and humans, even though the murine residue is always identical to the NMR residues in these positions. The PAPP-A cleavage sites in IGFBP-2, -4, and -5 [26–28] are all intact (Fig 4, S3 and S5 Figs). Due to the phenotypic similarities between NMRs and PAPP-A KO mice in terms of longevity and resistance to age-related diseases, PAPP-A was a target of particular interest.

There are conflicting results regarding the active site of PAPP-A [29], which is predicted to be mutated when analyzing the first available NMR genome [10], but is completely conserved in the other NMR genome sequence [30]. We, therefore, re-sequenced the exon containing the active site (exon 4) of NMR PAPP-A, using PCR and Sanger sequencing, and found no differences compared to the human PAPP-A active site sequence, and thus, no amino acid variations in the NMR active site of PAPP-A.

**Fig 5:**
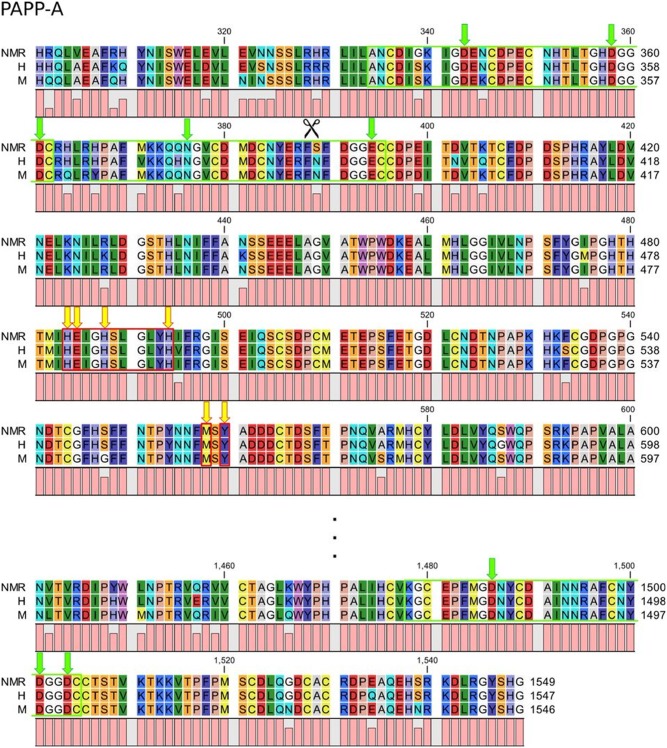
Sequence alignment of NMR, human, and mouse PAPP-A proteolytic domain, LNR1-3 modules, and the auto-proteolytic site. The LNR modules and the active site are enclosed within a green and red square, respectively. Amino acid residues necessary for proteolytic activity are indicated by yellow arrows in the active site and Met-turn, and by green arrows in each LNR module. The auto-cleavage site is indicated by scissors.

Despite the active site being intact in NMR PAPP-A, several other regions are required for proteolysis [31]. Met556, involved in a tight Met-turn, and possibly the adjacent Tyr557 residue are essential to the structural integrity of the active site environment, while the three lin12-notch repeat (LNR1-3) modules are required for proteolysis of IGFBP-4, but not IGFBP-5 [32]. They might also be relevant for IGFBP-2 cleavage. The Met-turn and close by tyrosine residue is 100% conserved in PAPP-A (Fig 5). In PAPP-A LNR1 [32], the NMR deviates from the human and murine sequences in position 339, where there is a glycine residue instead of serine. This may be a tolerated variation since it is not one of the more important residues that are involved in Ca^2+^-binding (indicated by green arrows). The same is true for LNR2, where the NMR PAPP-A only deviated from both the human and murine asparagine residue at position 389 (serine in the NMR sequence), but this is likewise not one of the indicated Ca^2+^-binding sites. We, therefore, predict that the proteolytic function most likely is retained despite these differences. However, the autoproteolytic site is disrupted by the variation in PAPP-A LNR2. It is difficult to interpret the physiological consequence of this variation, since it is not known to what degree autoproteolysis regulates PAPP-A in vivo. LNR3 is completely conserved between all three species.

### PAPP-A protein and proteolytic activity

**Fig 6:**
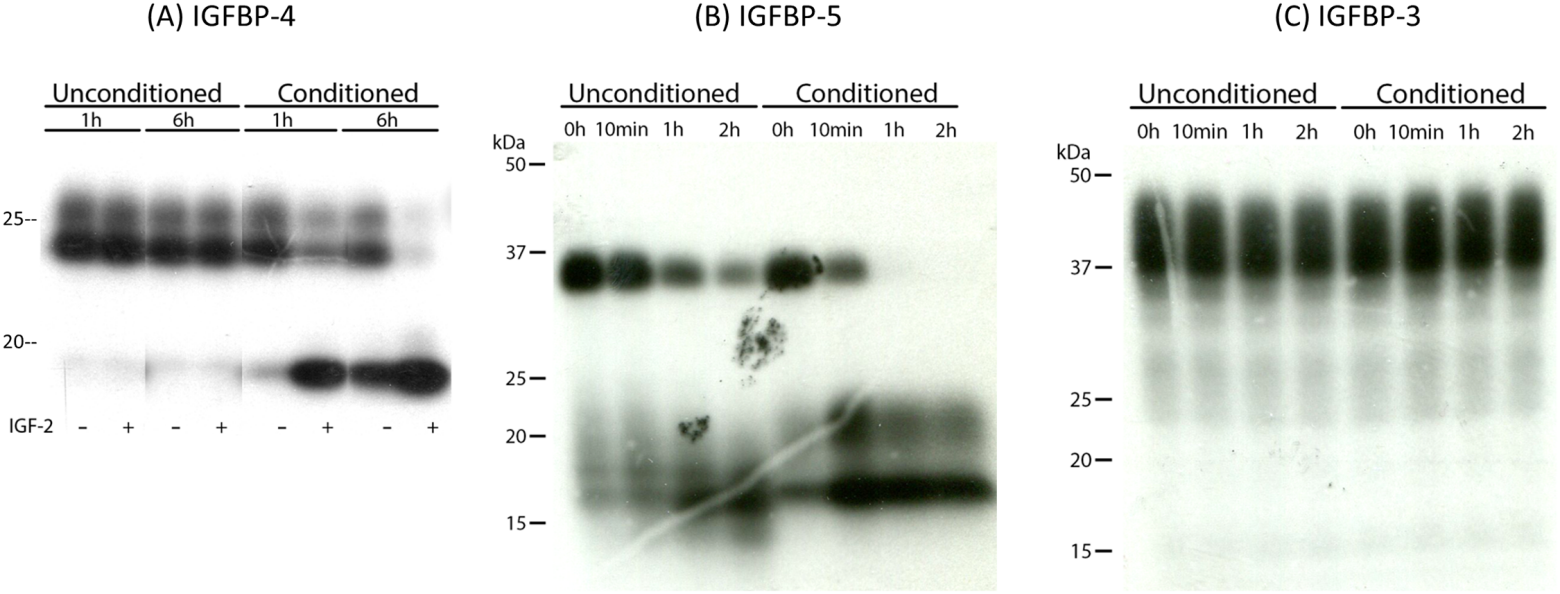
IGFBP proteolysis in conditioned medium from NMR fibroblasts. NSF CM or unconditioned SFM was incubated with (A) ^125^I-IGFBP-4 ± IGF-2 for 1 and 6 hours, (B) ^125^I-IGFBP-5 for 0, 10, 60, and 120 minutes, and (C) ^125^I-IGFBP-3 for 0, 10, 60, and 120 minutes.

PAPP-A protein secreted by NMR skin fibroblasts (NSFs) was detectable using a human PAPP-A ELISA. This ELISA does not recognize mouse PAPP-A, underscoring the high degree of identity between human and NMR PAPP-A. However, the question remained if the proteolytic activity was retained in the NMR. This was addressed by adding ^125^I-labeled human IGFBP-4, -5 or -3 to conditioned medium (CM) from cultured NSFs, followed by identification of proteolytic fragments by autoradiography. NSF CM contained an IGF-dependent IGFBP-4 cleavage activity (Fig 6A), strong support that the secreted NMR PAPP-A is an active IGFBP-4 protease and that PAPP-A can cleave IGFBP-4 in NMRs. Proteolytic activity towards IGFBP-5 was also apparent (Fig 6B). However, some time-dependent proteolysis also occurred in the unconditioned control medium as well, indicating a proteolytic contamination of the medium with IGFBP-5-cleaving capability. Regardless, complete proteolysis was observed between 10 and 60 minutes when adding IGFBP-5 to conditioned medium, while proteolysis was still incomplete after a 2-hour incubation of the IGFBP in unconditioned medium. Contrary to the proteolytic activity observed when adding exogenous IGFBP-4 and -5 to NSF CM, no considerable cleavage was evident when adding IGFBP-3 to the same medium (Fig 6C). This indicates a lack of IGFBP-3-cleaving proteases secreted by the NMR fibroblasts and also serves as a positive control for PAPP-A cleavage, since this protease does not cleave IGFBP-3.

### Differential regulation of PAPP-A and IGFBPs in the NMR

Although PAPP-A and IGFBP sequences are conserved between NMR and humans, the regulation of their expression could be different, thereby influencing IGF availability. Thus, we investigated the effects of growth factors, cytokines, and pharmacologic agents associated with aging, and with known effects in human cells, on mRNA levels in NMR fibroblasts, as assessed by real-time PCR. Findings are summarized in Table 2. Actual fold changes are presented if statistical significance was indicated by the method of Pfaffl. No regulation of PAPP-A expression in NSFs was apparent with stimulation by IGFs, insulin, tumor necrosis factor (TNF)-α, interleukin (IL)-6 (with soluble receptor), resveratrol or rapamycin. IL-1β had a small but significant effect to increase PAPP-A expression 1.7-fold. Transforming growth factor (TGF)-β had a slight inhibitory effect.

**Table 2:** Differential regulation of PAPP-A and IGFBPs in NMR fibroblasts.

	IGF-2	Insulin	LR3-IGF-1	TNF-α	IL-1β	TGF-β	IL-6+R	Resveratrol	Rapamycin
PAPP-A	**-**	**-**	**-**	**-**	**↑** x 1.67	**↓** x 0.90	**-**	**-**	**-**
IGFBP-2	**-**	**-**	**-**	**↑** x 1.64	**↑** x 2.07	**-**	**↑** x 1.89	**-**	**-**
IGFBP-3	**-**	**-**	**-**	**-**	**↑** x 1.38	**-**	**-**	**-**	**↑** x 1.66
IGFBP-4	**-**	**-**	**↑** x 1.20	**↑** x 1.50	**↑** x 4.00	**↓** x 0.61	**-**	**↑** x 2.73	**↑** x 2.45
IGFBP-5	**↑** x 2.56	**-**	**-**	**↑** x 1.46	**↑** x 2.23	**-**	**-**	**-**	**↓** x 0.80
IGFBP-6	**↑** x 1.55	**-**	**↑** x 1.34	**-**	**↑** x 1.50	**-**	**-**	**↓** x 0.85	**↓** x 0.85

Results are expressed as fold changes in expression levels of mRNA, as assessed by real-time PCR, following 24-hour stimulation with the indicated compounds. LR3-IGF-1 is an IGF-1 analog with markedly reduced affinity for IGFBPs. Down arrows indicate a significant down-regulation of the given mRNA with stimuli relative to control, whereas up arrows indicate a significant up-regulation. Bars signify no significant difference in mRNA expression between stimulated and unstimulated fibroblasts.

These data are in contrast to what has been found in several human cell culture studies. At the concentrations used in these experiments, both IL-1β and TNF-α were potent stimulators of PAPP-A expression in human fibroblasts, vascular smooth muscle cells, and preadipocytes [33–35]. It was not so surprising that there was a lack of a stimulatory effect of TGF-β or IL-6, since the effects of these agents are more cell-type specific, i.e., osteoblasts and vascular smooth muscle cells, respectively [31, 33]. Resveratrol has been shown to inhibit PAPP-A expression in human vascular smooth muscle cells and preadipocytes [34, 36], and postulated to mediate some of the health benefits seen with resveratrol administration to mice. However, resveratrol may work through preventing up-regulation of PAPP-A by pro-inflammatory cytokines [34], which was not tested in these studies in NSFs. Rapamycin was chosen due to its slowing effects on aging and age-related diseases via inhibition of the TOR kinase, which cross-talks with IGF signaling pathways [37]. There was no significant change in PAPP-A protein levels in the NSF CM with any of the treatments, except for a tendency for decreased levels in resveratrol-treated cultures (data not shown).

Apart from effects on PAPP-A, several of the regulatory compounds tested were capable of regulating the IGFBPs as well (Table 2). This is essential when interpreting data due to possible counteracting or enhancing effects. For instance, though PAPP-A is up-regulated in the presence of IL-1β, this may not result in the expected increase in IGF signaling because all of the IGFBPs were up-regulated simultaneously. Notable is the highest fold change of IGFBP-4 in response to IL-1β (4-fold), which is the primary physiological PAPP-A substrate.

**Fig 7:**
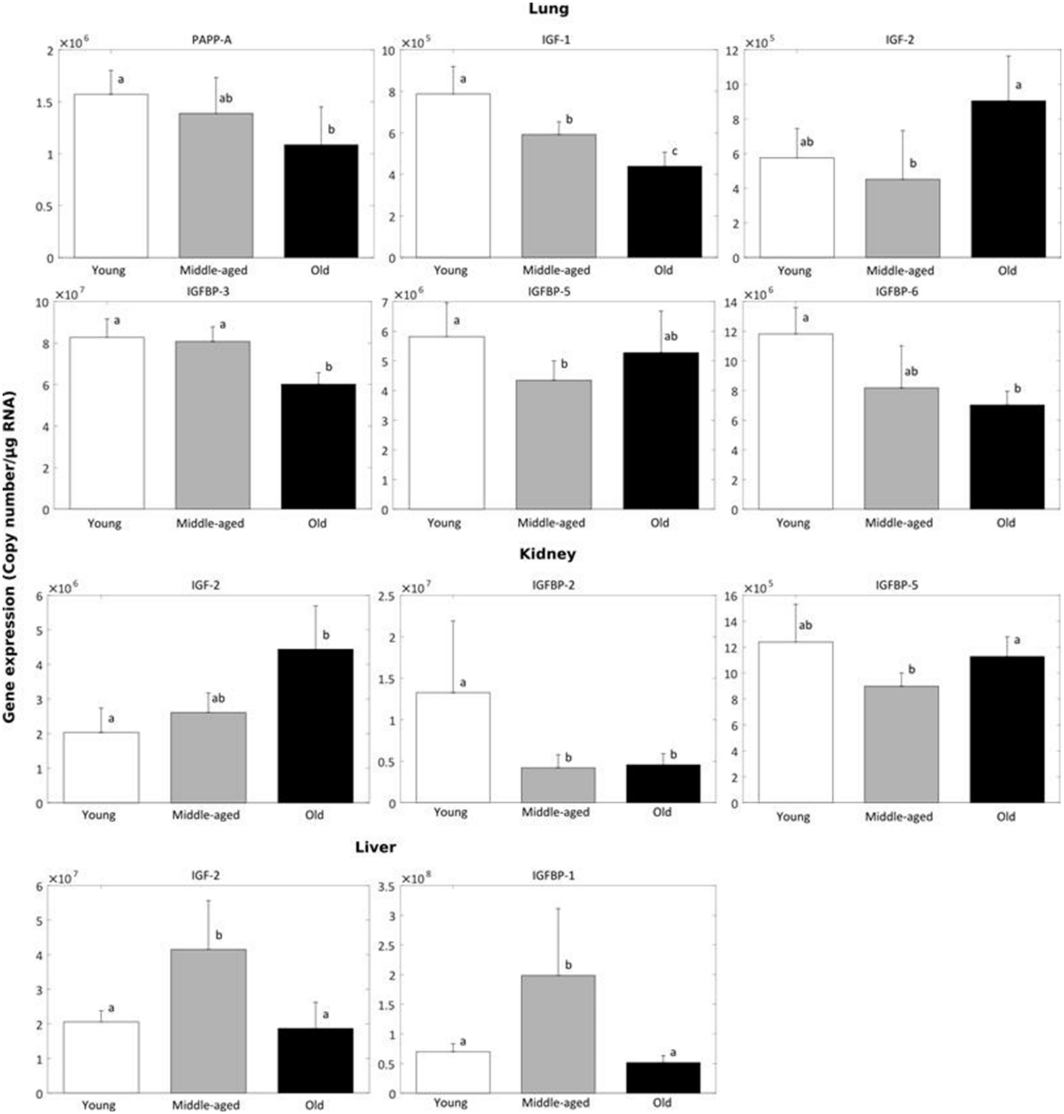
NMR tissue-specific changes in IGF system components with age. Changes in IGF-1 and -2, IGFBP-1 through -6, PAPP-A, and IGF-1 and -2 receptor mRNA levels were analyzed by real-time PCR in lung, liver and kidney of young (8–12 months), middle-age (4.5–6 years) and old (17–22 years) NMR (n = 4–5). Only data showing a significant up- or down-regulation with age are displayed. Groups not connected by the same letters are significantly different at *P* < 0.05.

Changes in the mRNA expression of the IGF system components in NMR tissues with age were also investigated. Kidney, liver, and lung from young (8–12 months), middle-aged (4.5–6 years) and old (17–22 years) NMRs were evaluated. Data from tissues and genes that showed significant changes with age are presented in Fig 7 (expression levels for all IGF system components are found in S1 Table).

Lung had the most changes with significant decreases in PAPP-A, IGF-1, and IGFBP-3, -5, and -6 expression with age. Lung IGF-2 was increased in old compared to middle-aged NMR. Kidney showed a progressive increase in IGF-2 expression with age, with a 2-fold increase in old NMR kidney compared to young. Age-related changes were also seen in NMR kidney IGFBP-2 and IGFBP-5 expression. Liver had a marked decrease in IGF-2 and IGFBP-1 expression in old and young compared to middle-aged NMR. The tissue–specific gene expression of the other components of the IGF system showed no significant difference across age, which could be due, in part, to the small sample size (4–5 NMR tissue samples). On the other hand, while up- or down-regulation of certain genes may be detrimental, an age-steady expression may indicate a healthy phenotype. Few tissue-specific and age-related studies have been performed. In the mouse, PAPP-A mRNA expression increased in kidney, brain and gonads, and decreased in bone and skeletal muscle, with age [38]. Mouse liver expressed very little PAPP-A. Unfortunately, lung was not examined in that study. Further studies are necessary, e.g., age-related comparisons with other species and additional tissues, to direct questions of relevance.

### Summary

There were no major variations occurring in the essential regions of IGF system gene sequences when comparing NMRs to humans and mice. Despite mutations present in the active site of NMR PAPP-A in the first available genome sequence, it was demonstrated here that the active site is indeed intact. Moreover, the protease is both expressed by and translated in NMR skin fibroblasts and retains IGF-dependent protease activity towards IGFBP-4 and IGF-independent activity towards IGFBP-5. Even though a natural PAPP-A knock-out phenomenon cannot account for NMR longevity, different regulation mechanisms of PAPP-A in NMR cells were observed both at the transcriptional and translational level compared to previous findings for human cells and for mouse tissues with age. Deviations from fine-tuned regulation of IGFBPs and their modulating proteases are likely to have major impact on physiology and pathophysiology.

This overall description of the IGF system in the NMR represents an important step towards elucidating the complex molecular mechanisms underlying longevity and how these animals have evolved to ensure a delayed and healthy aging process that may be applicable to humans.

## Experimental Procedures

### Gene annotation

In order to enable comparison of human, murine, and NMR sequences belonging to the IGF system, annotation of NMR IGF components was carried out using the BLAT search tool from the UCSC Genome Browser (Genome Accession: AFSB00000000 and AHKG00000000). The six-frame translated NMR genome databases were searched using human and murine protein queries (S2 Table), thereby avoiding the degeneracy of the genetic code resulting in mismatched nucleotides despite identical amino acid outcome.

The BLAT search results were supported by downloading both available NMR genomes, importing them into CLC workbench, and using the tBLASTn tool (Gap cost = 7, Extension cost = 2, Matrix = BLOSOM62) with identical query sequence input as above. Where missing, start methionines and stop codons were looked for manually in the ends of the first and last exon, respectively. Following annotation, all genes were translated into protein, whereupon inter-species protein alignment analyses were carried out using the CLC Sequence Viewer alignment tool (Gap cost = 10. Extension cost = 1).

### PAPP-A exon 4

Primers for amplification of exon 4 from NMR PAPP-A were designed using Primer3 (fwd: 5’- ACTTGGCATTTTTGCACCAG-3’, rev: 5’-TCAAGGTTACCCCGAGTCAG-3’, amplicon = 452 bp), and their specificity was verified using the NCBI BLAST tool comparing each primer against the complete NMR genome. Purification of PAPP-A exon 4 was carried out using the NucleoSpin^®^ Extract II method (Qiagen, Netherlands), and the purified product verified on a 1.5% agarose gel.

### Cell-based experiments

NMR skin fibroblasts (NSFs) were cultured in polystyrene-surface cell culture flasks (Corning) and nourished with Eagle’s Minimal Essential Medium (EMEM) supplemented with 15% Fetal Bovine Serum (FBS) (Gibco, certified) and 1% PenStrep (100 units/mL penicillin, 100 g/mL streptomycin) (Gibco), as previously described [39]. At all times, the NSFs were incubated at 3% O_2_, 5% CO_2_, and 32°C in a CO_2_ water-jacketed incubator. For experiments, NSFs were washed and changed to serum-free medium (SFM; 0.1% BSA and 1% PenStrep in EMEM) with various treatments. At the end of the indicated incubation time, conditioned medium (CM) was collected for protein and proteolytic assays and cells harvested in TRIzol for mRNA expression analyses.

### Enzyme-linked immunosorbent assay

PAPP-A ELISA was carried out on NSF CM using a human picoPAPP-A ELISA kit, generously provided by AnshLabs (Webster, TX).

### Protease assays

PAPP-A protease assays for ^125^I-labeled IGFBPs were performed on NSF CM as previously described [36], except the incubation temperature was adjusted to 32°C in order to imitate the natural body temperature of the NMR. For the IGF-dependent PAPP-A-mediated proteolysis of IGFBP-4 [27, 40], IGF-2 was added to the cell-free reaction mixture.

### Real-time PCR

Primers for real-time PCR (S3 Table) were designed using Primer3 and the annotated NMR mRNA sequences. These sequences were pre-adjusted as to avoid primers to be situated in ambiguous regions such as exon-exon junctions as well as to make them intron-spanning. The theoretical specificity of the primers was examined using the CLC workbench BLAST tool comparing each primer against both available NMR genomes.

Total RNA was purified from liver, kidney and lungs from young (8–12 months), middle–aged (4.5–6 years) and old (17–22 years) NMRs as well as from NSFs, as previously described [34], and final concentrations were determined using a ND1000 NanoDrop spectrophotometer (Thermo Scientific, USA). One μg RNA was reverse transcribed with SuperScript^™^ III RT enzyme (Invitrogen). The actual specificity of the designed primer sets was tested by end-point PCR. Real-time PCR was performed using the iCycler iQ5 Detection System with iQ SYBR Green PCR Master Mix (BioRad), as previously described [38]. Data were analyzed with the Pfaffl method [41] using the REST 2009 v2.0.13 program.

## Supporting Information

S1 Fig Sequence alignment of NMR, human, and mouse IGF-2R (domains 11–13). Grey arrows indicate residues interacting with IGF-2.
(TIF)

S2 Fig Sequence alignment of NMR, human, and mouse IGFBP-1. NMR sequence A is annotated based on genome accession AFSB00000000, whereas NMR sequence B is annotated based on genome accession AHKG00000000. The N-terminal GCGCC motif is enclosed within a red square. The high-affinity N-terminal binding site is enclosed within a black square. Phosphorylation sites are indicated by a red P.
(TIF)

S3 Fig Sequence alignment of NMR, human, and mouse IGFBP-2. The N-terminal GCGCC motif is enclosed within a red square. The high-affinity N-terminal binding site is enclosed within a black square. RGD motifs are enclosed within a green square. HBDs are enclosed within an orange square. The PAPP-A proteolytic site is indicated by scissors.
(TIF)

S4 Fig Sequence alignment of NMR, human, and mouse IGFBP-3. The N-terminal GCGCC motif is enclosed within a red square. The high-affinity N-terminal binding site is enclosed within a black square. Phosphorylation sites are indicated by a red P. HBDs are enclosed within an orange square. Major basic domains are enclosed within a yellow square. Glycosylation sites are indicated with a sugar branch.
(TIF)

S5 Fig Sequence alignment of NMR, human, and mouse IGFBP-5. The N-terminal GCGCC motif is enclosed within a red square. The high-affinity N-terminal binding site is enclosed within a black square. HBDs are enclosed within an orange square. Major basic domains are enclosed within a yellow square. Glycosylation sites are indicated with a sugar branch. The PAPP-A proteolytic site is indicated by scissors.
(TIF)

S6 Fig Sequence alignment of NMR, human, and mouse IGFBP-6. The N-terminal GCGCC motif is enclosed within a red square. The high-affinity N-terminal binding site is enclosed within a black square. HBDs are enclosed within an orange square. Major basic domains are enclosed within a yellow square. Glycosylation sites are indicated with a sugar branch.
(TIF)

S1 Table Overview of the expression levels of IGF system components in NMR kidney, liver, and lung tissue. Numbers not connected by the same letter are significantly different (p < 0.05).
(DOCX)

S2 Table Query sequences for gene annotation. (DOCX)

S3 Table Primer sequences for real time PCR. (DOCX)
