# Supplementary figures and images for: The Insulin-Like Growth Factor System in the Long-Lived Naked Mole-Rat
Source: PLoS One. 2015 Dec 22;10(12):e0145587. doi: 10.1371/journal.pone.0145587 (PMC4694111; doi:10.1371/journal.pone.0145587)

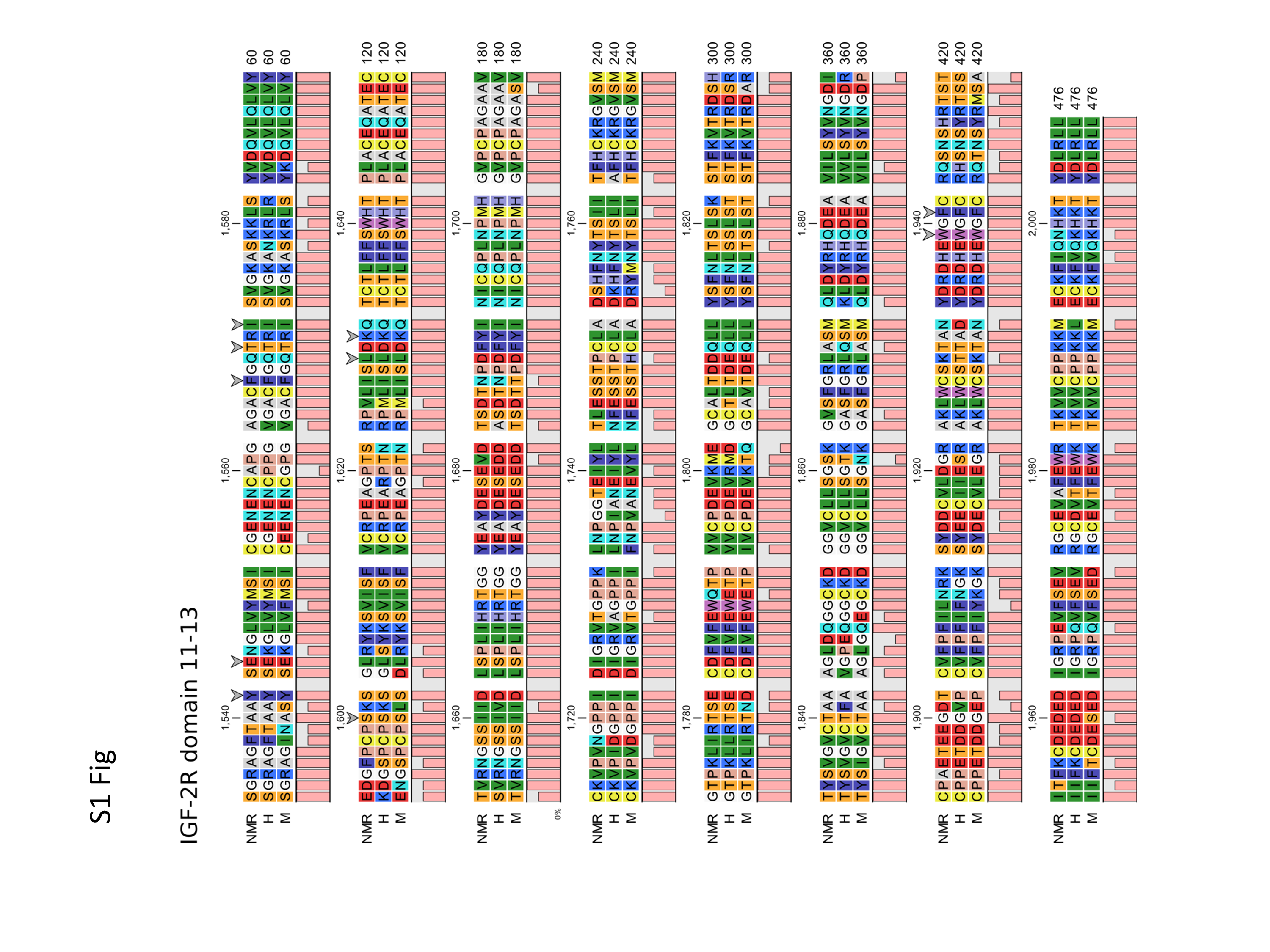

Supplement: S1 Fig — Grey arrows indicate residues interacting with IGF-2. (TIF) [file pone.0145587.s001.TIF]

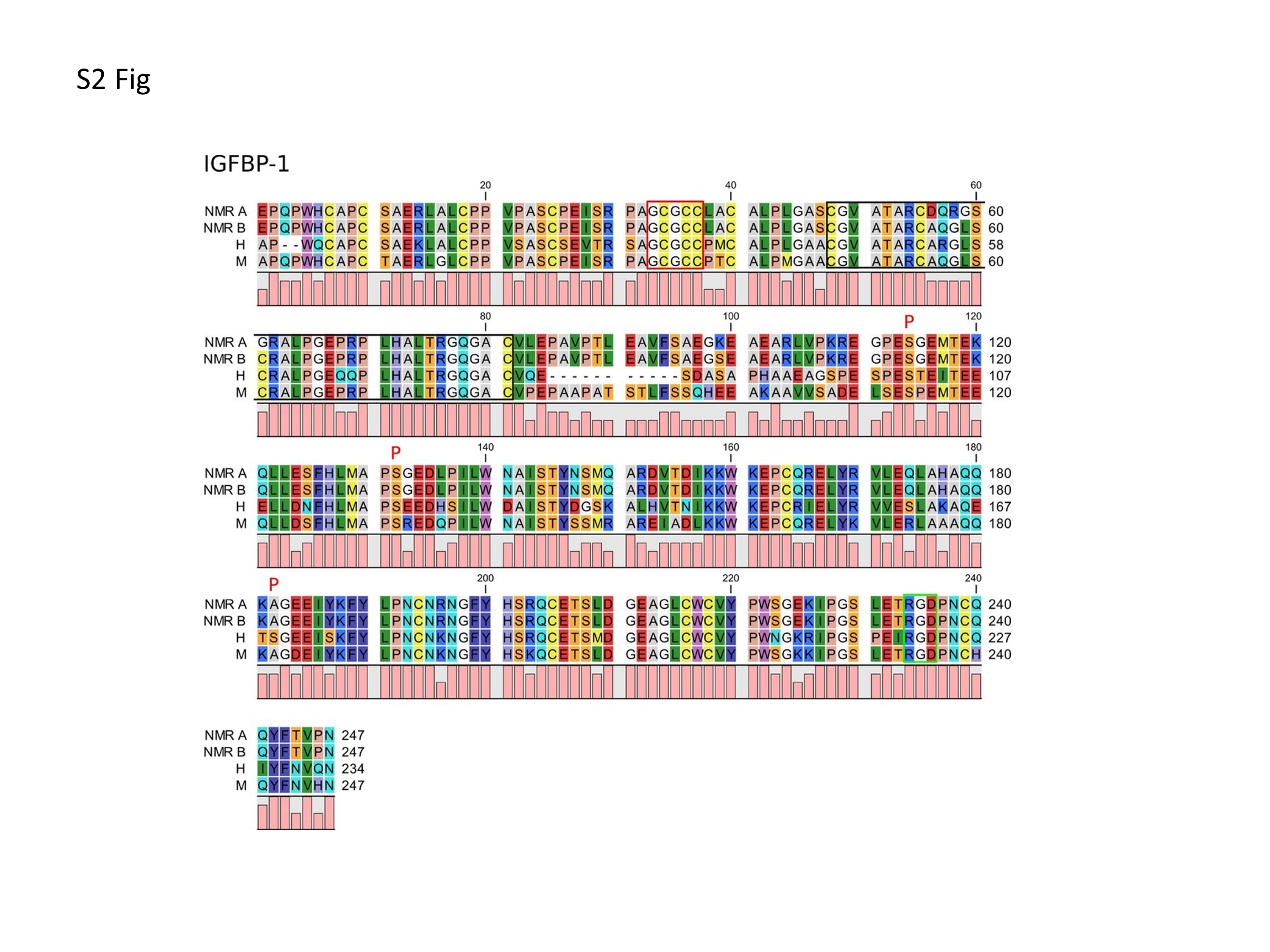

Supplement: S2 Fig — NMR sequence A is annotated based on genome accession AFSB00000000, whereas NMR sequence B is annotated based on genome accession AHKG00000000. The N-terminal GCGCC motif is enclosed within a red square. The high-affinity N-terminal binding site is enclosed within a black square. Phosphorylation sites are indicated by a red P. (TIF) [file pone.0145587.s002.TIF]

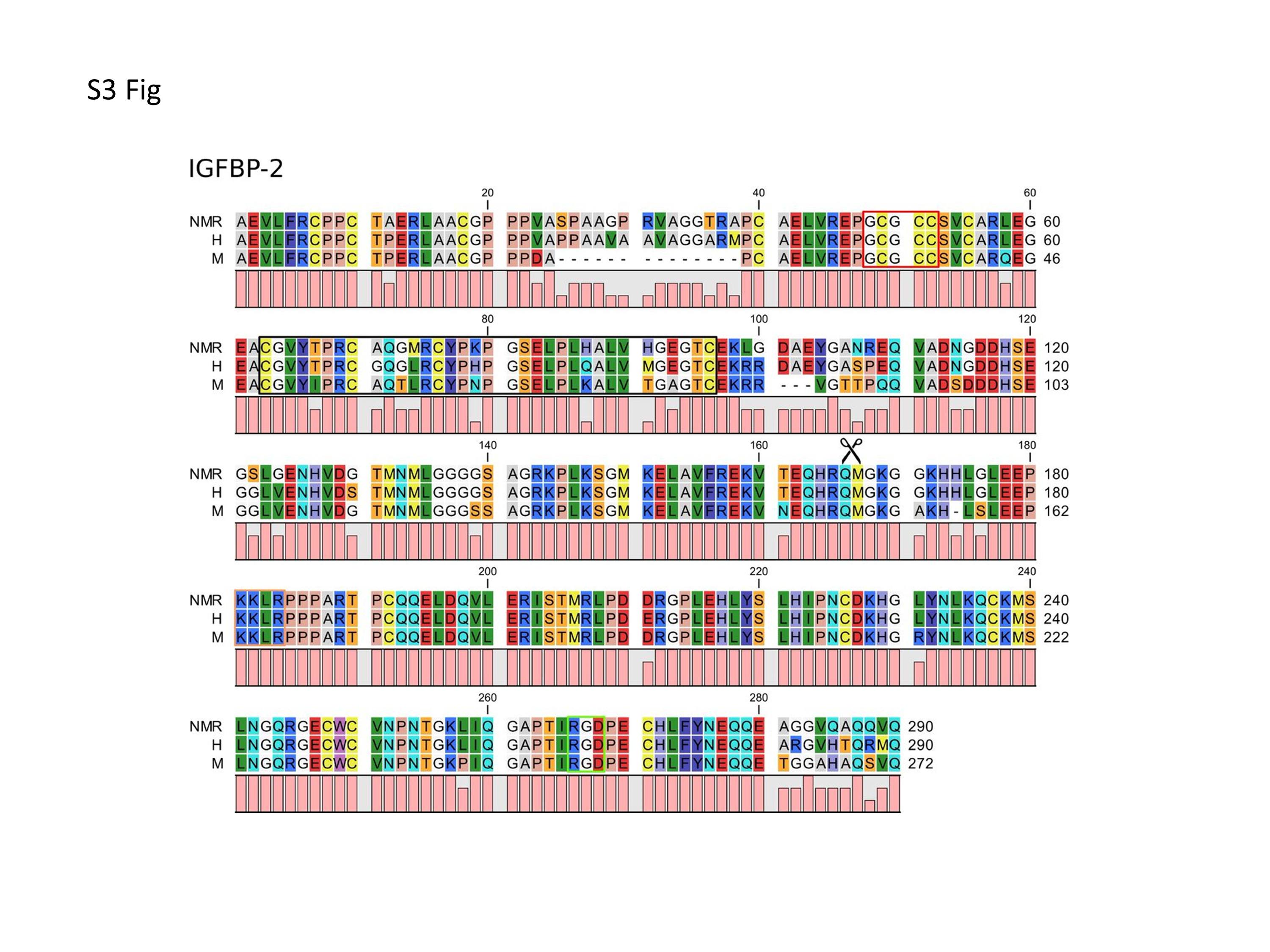

Supplement: S3 Fig — The N-terminal GCGCC motif is enclosed within a red square. The high-affinity N-terminal binding site is enclosed within a black square. RGD motifs are enclosed within a green square. HBDs are enclosed within an orange square. The PAPP-A proteolytic site is indicated by scissors. (TIF) [file pone.0145587.s003.TIF]

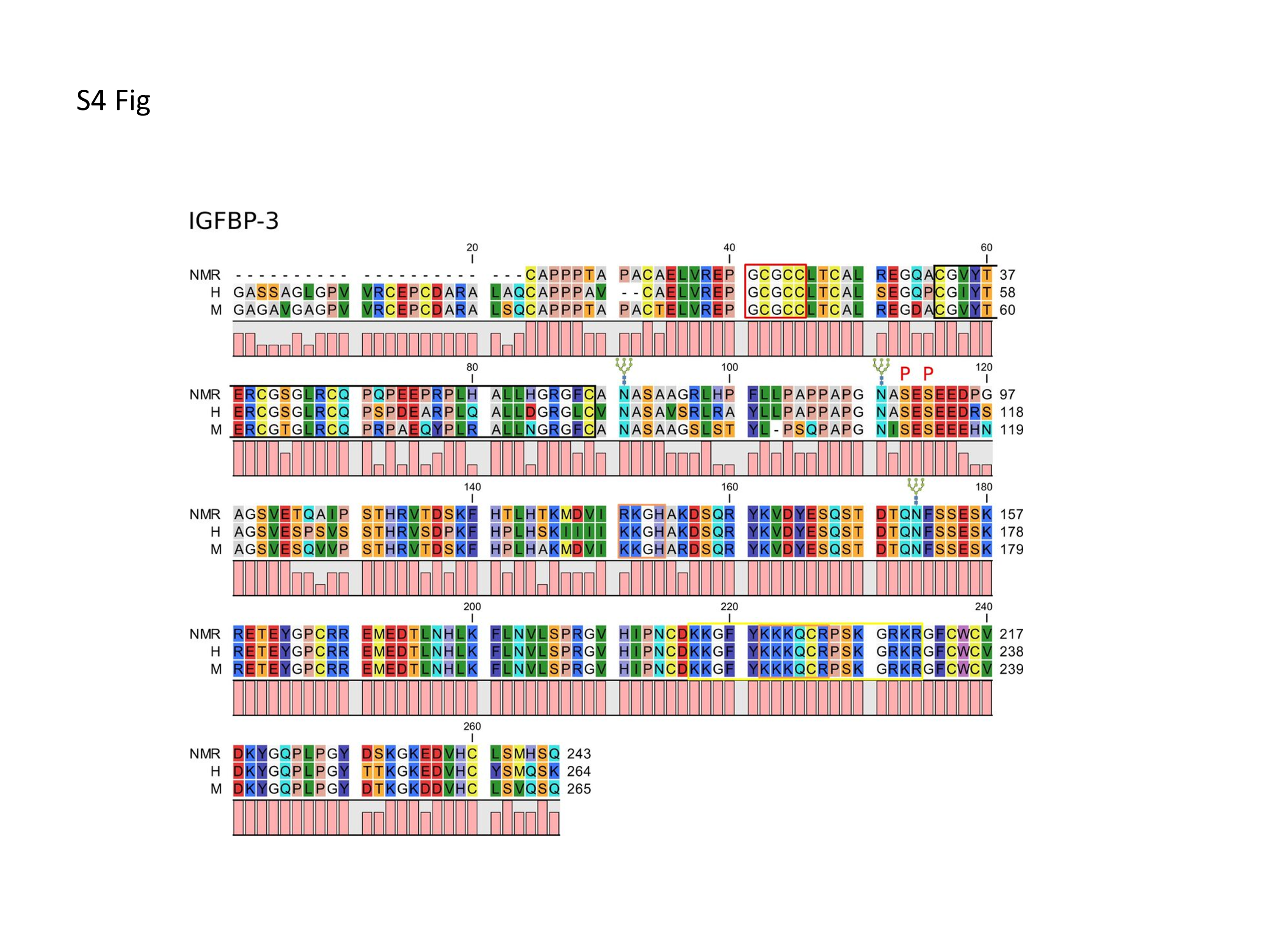

Supplement: S4 Fig — The N-terminal GCGCC motif is enclosed within a red square. The high-affinity N-terminal binding site is enclosed within a black square. Phosphorylation sites are indicated by a red P. HBDs are enclosed within an orange square. Major basic domains are enclosed within a yellow square. Glycosylation sites are indicated with a sugar branch. (TIF) [file pone.0145587.s004.TIF]

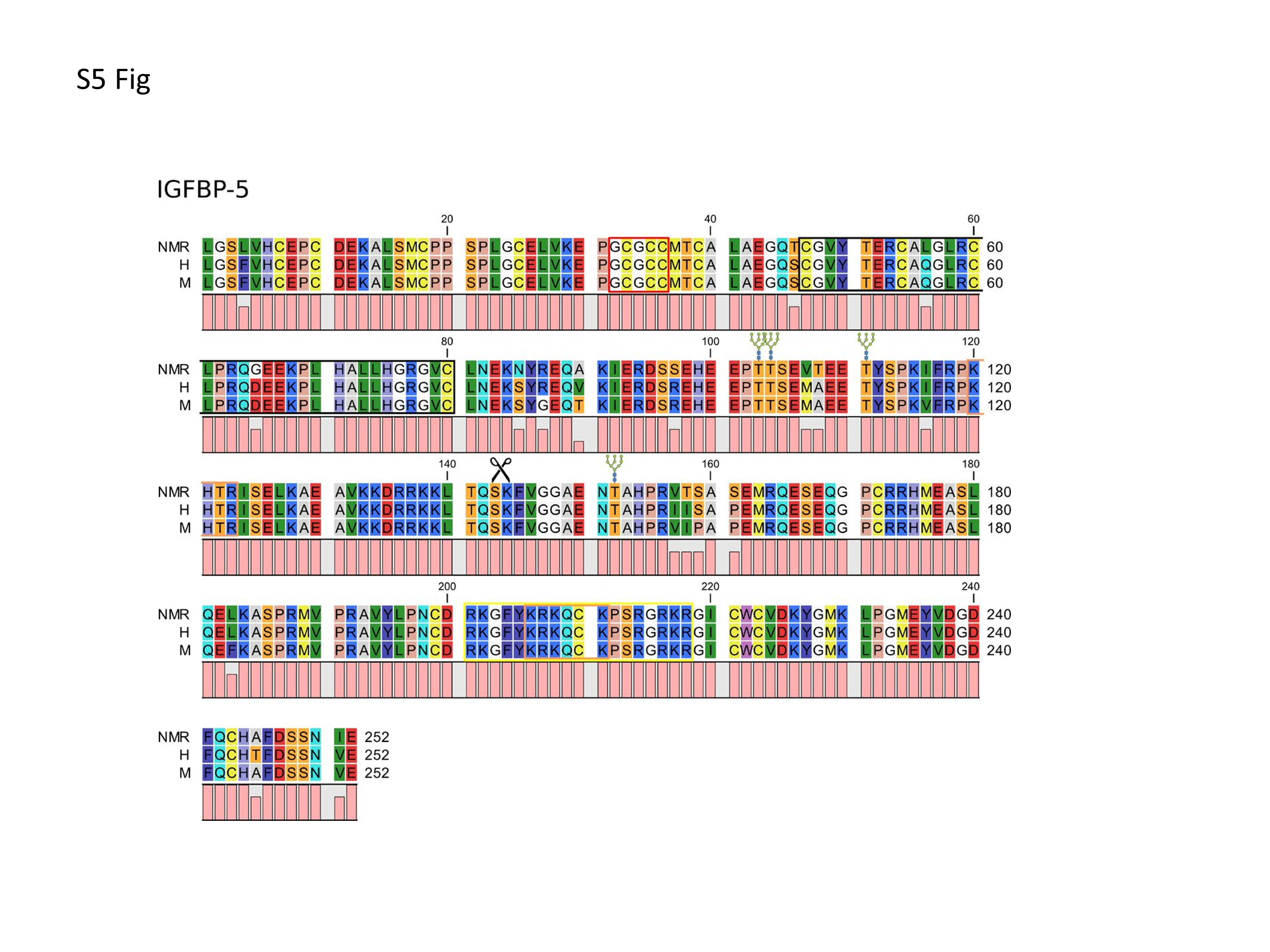

Supplement: S5 Fig — The N-terminal GCGCC motif is enclosed within a red square. The high-affinity N-terminal binding site is enclosed within a black square. HBDs are enclosed within an orange square. Major basic domains are enclosed within a yellow square. Glycosylation sites are indicated with a sugar branch. The PAPP-A proteolytic site is indicated by scissors. (TIF) [file pone.0145587.s005.TIF]

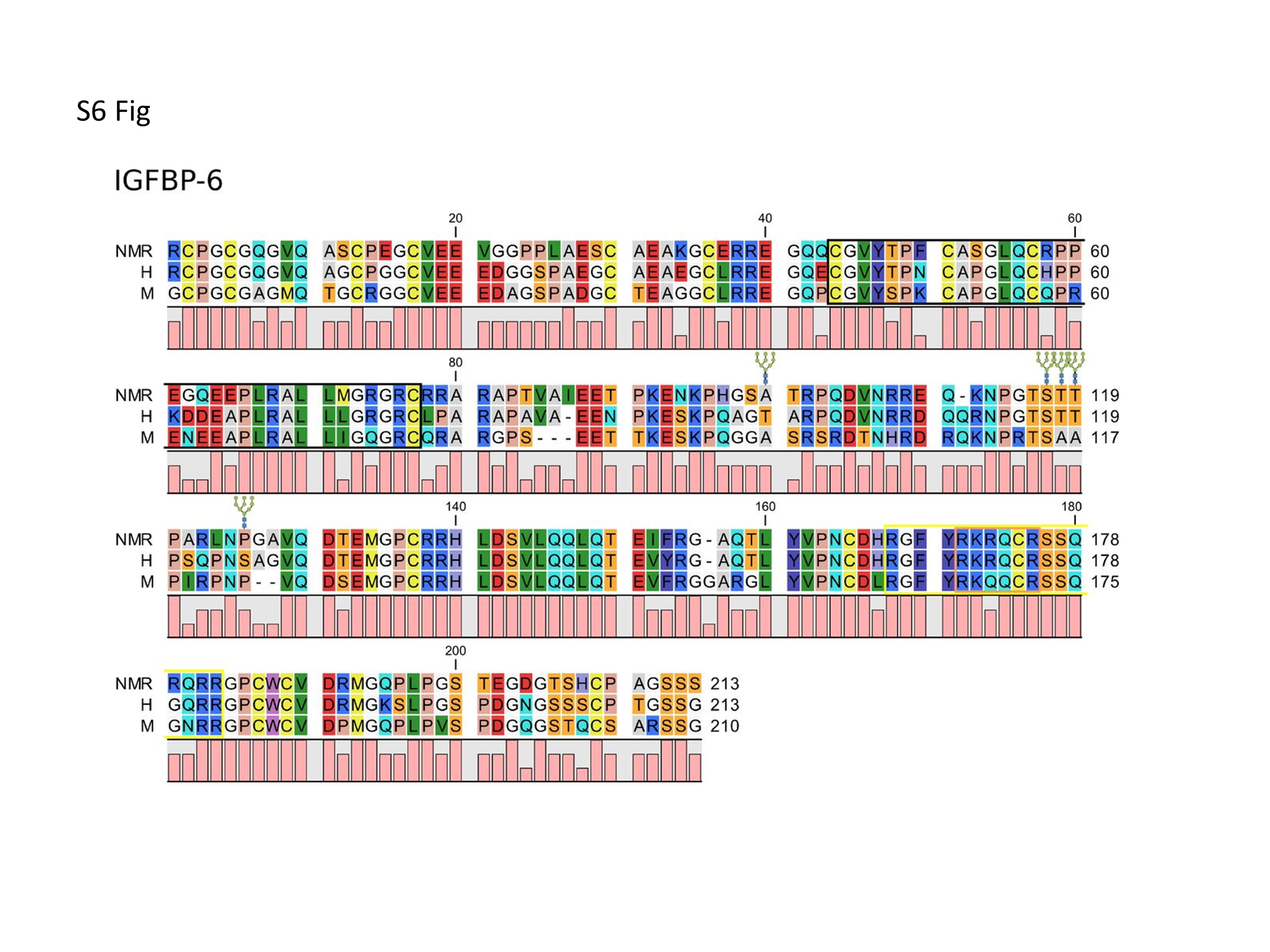

Supplement: S6 Fig — The N-terminal GCGCC motif is enclosed within a red square. The high-affinity N-terminal binding site is enclosed within a black square. HBDs are enclosed within an orange square. Major basic domains are enclosed within a yellow square. Glycosylation sites are indicated with a sugar branch. (TIF) [file pone.0145587.s006.TIF]
