# Supplementary material for: The Insulin-Like Growth Factor System in the Long-Lived Naked Mole-Rat
Source: PLoS One. 2015 Dec 22;10(12):e0145587. doi: 10.1371/journal.pone.0145587 (PMC4694111; doi:10.1371/journal.pone.0145587)
Supplement: S1 Table — Numbers not connected by the same letter are significantly different (p < 0.05). (DOCX) [file pone.0145587.s007.docx]

**S1 Table. Overview of the expression levels of IGF system components in NMR kidney, liver, and lung tissue.**

|  | Young | | Middle aged | | Old | |
| --- | --- | --- | --- | --- | --- | --- |
|  | **Kidney** | | | | | |
|  | Copy #/ug RNA | SE | Copy #/ug RNA | SE | Copy #/ug RNA | SE |
| IGF-1 | 3.0^5^ | 5.4^4^ | 3.0^5^ | 8.5^4^ | 2.7^5^ | 1.9^4^ |
| IGF-2 | ^a^2.0^6^ | 3.5^5^ | ^ab^2.6^6^ | 2.5^5^ | ^b^4.4^6^ | 6.3^5^ |
| IGFBP-1 | 5.0^7^ | 5.0^6^ | 5.6^7^ | 4.3^6^ | 6.3^7^ | 7.0^6^ |
| IGFBP-2 | ^a^1.3^7^ | 4.3^6^ | ^b^4.2^6^ | 7.0^5^ | ^b^4.6^6^ | 6.0^5^ |
| IGFBP-3 | 4.2^7^ | 9.5^6^ | 4.5^7^ | 5.1^6^ | 5.2^7^ | 6.1^6^ |
| IGFBP-4 | 8.5^5^ | 1.9^5^ | 5.0^5^ | 3.8^4^ | 5.9^5^ | 1.1^5^ |
| IGFBP-5 | ^ab^1.2^6^ | 1.5^5^ | ^b^9.0^5^ | 5.1^4^ | ^a^1.1^6^ | 6.8^4^ |
| IGFBP-6 | 1.6^7^ | 3.3^6^ | 1.4^7^ | 1.2^6^ | 2.0^7^ | 3.4^6^ |
| IGF-1R | 4.0^6^ | 4.7^5^ | 5.0^6^ | 6.0^5^ | 5.3^6^ | 5.4^5^ |
| IGF-2R | 6.2^6^ | 6.9^5^ | 6.1^6^ | 4.6^5^ | 8.1^6^ | 1.0^6^ |
| PAPP-A | 9.0^5^ | 1.7^5^ | 7.8^5^ | 6.7^4^ | 8.0^5^ | 3.1^4^ |
|  | **Liver** | | | | | |
|  | Copy #/ug RNA | SE | Copy #/ug RNA | SE | Copy #/ug RNA | SE |
| IGF-1 | 1.5^7^ | 1.6^6^ | 1.4^7^ | 7.6^5^ | 2.2^7^ | 5.2^6^ |
| IGF-2 | ^a^2.1^7^ | 1.5^6^ | ^b^4.2^7^ | 6.3^6^ | ^a^1.9^7^ | 3.4^6^ |
| IGFBP-1 | ^a^7.0^7^ | 7.8^6^ | ^b^2.0^8^ | 5.0^7^ | ^a^5.2^7^ | 5.3^6^ |
| IGFBP-2 | 5.8^7^ | 4.8^6^ | 4.5^7^ | 4.8^6^ | 6.6^7^ | 1.4^7^ |
| IGFBP-3 | 1.3^8^ | 1.4^7^ | 1.4^8^ | 1.2^7^ | 1.1^8^ | 4.4^6^ |
| IGFBP-4 | 6.5^6^ | 3.0^5^ | 5.8^6^ | 6.4^5^ | 6.2^6^ | 4.3^5^ |
| IGFBP-5 | 1.3^5^ | 2.9^4^ | 1.7^5^ | 6.2^4^ | 1.7^5^ | 2.2^4^ |
| IGFBP-6 | 1.1^6^ | 2.1^5^ | 1.3^6^ | 3.7^5^ | 9.8^5^ | 4.9^4^ |
| IGF-1R | 3.7^5^ | 4.6^4^ | 4.3^5^ | 6.1^4^ | 4.1^5^ | 6.7^4^ |
| IGF-2R | 7.9^6^ | 5.0^5^ | 6.3^6^ | 6.7^5^ | 7.9^6^ | 8.5^5^ |
| PAPP-A | 9.6^4^ | 2.1^4^ | 1.2^5^ | 2.5^4^ | 7.4^4^ | 9.6^3^ |
|  | **Lung** | | | | | |
|  | Copy #/ug RNA | SE | Copy #/ug RNA | SE | Copy #/ug RNA | SE |
| IGF-1 | ^a^7.9^5^ | 5.9^4^ | ^b^5.9^5^ | 2.8^4^ | ^c^4.4^5^ | 3.0^4^ |
| IGF-2 | ^ab^5.8^5^ | 8.5^4^ | ^b^4.5^5^ | 1.3^5^ | ^a^9.1^5^ | 1.3^5^ |
| IGFBP-1 | 9.0^6^ | 1.1^6^ | 8.6^6^ | 6.8^5^ | 6.3^6^ | 1.1^6^ |
| IGFBP-2 | 2.4^7^ | 2.1^6^ | 2.1^7^ | 1.8^6^ | 1.6^7^ | 2.2^6^ |
| IGFBP-3 | ^a^8.3^7^ | 4.0^6^ | ^a^8.1^7^ | 3.1^6^ | ^b^6.0^7^ | 2.5^6^ |
| IGFBP-4 | 5.0^6^ | 3.2^6^ | 1.8^6^ | 1.6^5^ | 1.7^6^ | 1.8^5^ |
| IGFBP-5 | ^a^5.8^6^ | 5.1^5^ | ^b^4.3^6^ | 2.9^5^ | ^ab^5.3^6^ | 7.0^5^ |
| IGFBP-6 | ^a^1.2^7^ | 8.0^5^ | ^ab^8.2^6^ | 1.3^6^ | ^b^7.0^6^ | 4.1^5^ |
| IGF-1R | 5.2^6^ | 4.9^5^ | 5.1^6^ | 1.0^6^ | 5.0^6^ | 7.5^5^ |
| IGF-2R | 7.1^6^ | 7.5^5^ | 7.2^6^ | 4.0^5^ | 8.7^6^ | 1.4^6^ |
| PAPP-A | ^a^1.6^6^ | 1.0^5^ | ^ab^1.4^6^ | 2.0^5^ | ^b^1.1^6^ | 1.6^5^ |

Numbers not connected by the same letter are significantly different (p < 0.05).
