# Supplementary material for: The Insulin-Like Growth Factor System in the Long-Lived Naked Mole-Rat
Source: PLoS One. 2015 Dec 22;10(12):e0145587. doi: 10.1371/journal.pone.0145587 (PMC4694111; doi:10.1371/journal.pone.0145587)
Supplement: S2 Table — (DOCX) [file pone.0145587.s008.docx]

**S2 Table. Query sequences for gene annotation**

| Name | Accession no. (murine) | Accession no. (human) |
| --- | --- | --- |
| IGF-1 | Q4VJB9 | E9PD02 |
| IGF-2 | P09535 | P01344 |
| IGFBP-1 | P47876 | P08833 |
| IGFBP-2 | P47877 | P18065 |
| IGFBP-3 | Q6PE62 | P17936 |
| IGFBP-4 | P47879 | P22692 |
| IGFBP-5 | Q07079 | P24593 |
| IGFBP-6 | P47880 | P24592 |
| PAPP-A | Q8R4K8 | Q13219 |
| IGF-1R | Q60751 | P08069 |
| IGF-2R | Q07113 | P11717 |
