# Supplementary material for: The Insulin-Like Growth Factor System in the Long-Lived Naked Mole-Rat
Source: PLoS One. 2015 Dec 22;10(12):e0145587. doi: 10.1371/journal.pone.0145587 (PMC4694111; doi:10.1371/journal.pone.0145587)
Supplement: S3 Table — (DOCX) [file pone.0145587.s009.docx]

**S3 Table. Primer sequences for real time PCR.**

| Naked mole rat gene | Primer sequence 5’-3’ | Product size |
| --- | --- | --- |
| Igf1  Forward | TGTCCTTTTCGCACCTCTTC | 171 bp |
| Reverse | GACTGCTGGAGCCGTACC |  |
| Igf2  Forward | AAGTCCGAGAGGGACGTGT | 171 bp |
| Reverse | CTCCCTGGAGAGCATACGAC |  |
| Igfbp1  Forward | CCGAGACGTCACAGACATCA | 209 bp |
| Reverse | GGTAGACGCACCAGCACAG |  |
| Igfbp2  Forward | AGAAAGTCACCGAGCAGCAC | 153 bp |
| Reverse | GAAGTCGCATGGTGGAGATT |  |
| Igfbp3  Forward | GACAGCCAGCGCTACAAAGT | 140 bp |
| Reverse | TTGAGGAACTTCAGGTGGTTG |  |
| Igfbp4  Forward | CATGGAGCCTGCGGAGAT | 84 bp |
| Reverse | AAGCTGTTGTTGGGATGCTC |  |
| Igfbp5  Forward | GAAAAGAATTACCGCGAGCA | 145 bp |
| Reverse | CTCAGAGATGCGAGTGTGCT |  |
| Igfbp6  Forward | TGACCATCGAGGCTTCTACC | 83 bp |
| Reverse | TTCGATCCACACACCAACAG |  |
| Pappa  Forward | GCCCAGATGGCTACTCCATA | 121 bp |
| Reverse | GTTGGGTTCAGCCAGTAAGG |  |
| Igf1r  Forward | ATGTCCTTTCGGCATCAAAC | 201 bp |
| Reverse | CCTCTTCAACGTCAATGGTG |  |
| Igf2r  Forward | ATGCACGCTCTTCTTCTCCT | 132 bp |
| Reverse | GAGGTGTCGACCTCACTTTCA |  |
| Tbp  Forward | TTGGAGGGTCTCGTGCTTAC | 90 bp |
| Reverse | TCTGGGTTTGATCATTCGGTA |  |
| Actb  Forward | GATCACCATCGGCAATGAG | 104 bp |
| Reverse | TGGAGTTGAAGGTAGTTTCGTG |  |
| Rpl19  Forward | AACCGTCACGGATGATGTG | 124 bp |
| Reverse | AACCGTCACGGATGATGTG |  |
